# Supplementary material for: Cutaneous Complications of mRNA and AZD1222 COVID-19 Vaccines: A Worldwide Review
Source: Microorganisms. 2022 Mar 15;10(3):624. doi: 10.3390/microorganisms10030624 (PMC8953728; doi:10.3390/microorganisms10030624)
Supplement: Supplementary file 1 [file microorganisms-10-00624-s001.zip › microorganisms-1516995-supplementary.pdf]

microorganisms

Supplementary materials

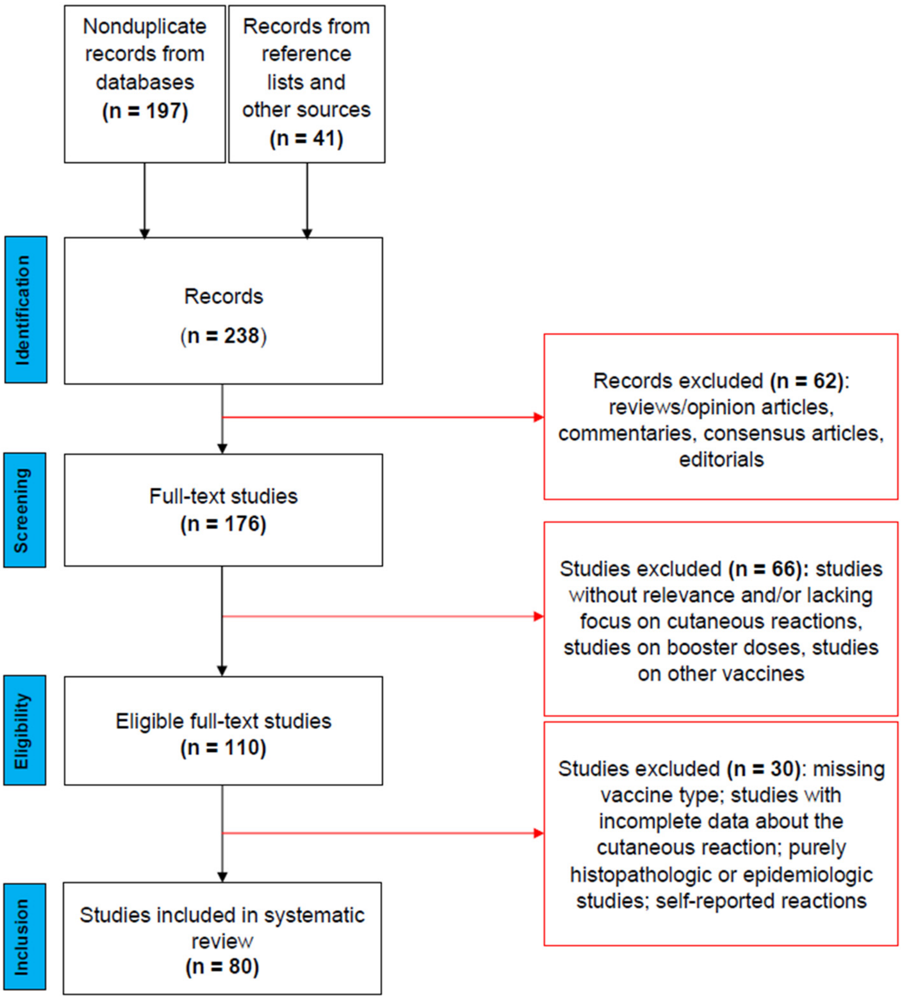

Figure S1. Flow diagram of study selection performed according to Preferred Reporting Items for Systematic Reviews and Meta-Analysis guidelines.
